# Unveiling a Shield of Hope: A Novel Multiepitope-Based Immunogen for Cross-Serotype Cellular Defense against Dengue Virus

**DOI:** 10.3390/vaccines12030316

**Published:** 2024-03-16

**Authors:** Nilanshu Manocha, Daphné Laubreton, Xavier Robert, Jacqueline Marvel, Virginie Gueguen-Chaignon, Patrice Gouet, Prashant Kumar, Madhu Khanna

**Affiliations:** 1 Amity Institute of Virology & Immunology, Amity University Uttar Pradesh, Sector-125, Noida 201313, Uttar Pradesh, India; nilanshu.manocha@student.amity.edu (N.M.); pkumar18@amity.edu (P.K.); 2 Centre International de Recherche en Infectiologie, Université de Lyon, INSERM U1111, CNRS UMR 5308, Ecole Normale Supérieure de Lyon, Université Claude Bernard Lyon 1, 69364 Lyon, France; daphne.laubreton@inserm.fr (D.L.); jacqueline.marvel@inserm.fr (J.M.); 3 Virology Unit, Department of Microbiology, Vallabhbhai Patel Chest Institute, University of Delhi, Delhi 110007, Delhi, India; 4 Molecular Microbiology and Structural Biochemistry, UMR 5086 CNRS Université de Lyon, 7 Passage du Vercors, CEDEX 07, 69367 Lyon, France; xavier.robert@ibcp.fr (X.R.); patrice.gouet@ibcp.fr (P.G.); 5 Protein Science Facility, Université Claude Bernard Lyon 1, CNRS UAR3444, INSERM US8, Ecole Normale Supérieure de Lyon, SFR Biosciences, 69007 Lyon, France; virginie.gueguen-chaignon@ibcp.fr

**Keywords:** dengue virus, immunization, cross-serotype protection, cellular immunity, multiepitope vaccine, MHC I-binding epitopes, memory T cells, vaccine candidate

## Abstract

Dengue virus (DENV) infection continues to be a public health challenge, lacking a specific cure. Vaccination remains the primary strategy against dengue; however, existing live-attenuated vaccines display variable efficacy across four serotypes, influenced by host serostatus and age, and predominantly inducing humoral responses. To address this limitation, this study investigates a multiepitope-based immunogen designed to induce robust cellular immunity across all DENV serotypes. The chimeric immunogen integrates H-2^d^ specific MHC-I binding T-cell epitopes derived from conserved domains within the DENV envelope protein. Immuno-informatics analyses supported its stability, non-allergenic nature, and strong MHC-I binding affinity as an antigen. To assess the immunogenicity of the multiepitope, it was expressed in murine bone-marrow-derived dendritic cells (BMDCs) that were used to prime mice. In this experimental model, simultaneous exposure to T-cell epitopes from all four DENV serotypes initiated distinct IFNγ-CD8 T-cell responses for different serotypes. These results supported the potential of the multiepitope construct as a vaccine candidate. While the optimization of the immunogen design remains a continuous pursuit, this proof-of-concept study provides a starting point for evaluating its protective efficacy against dengue infection in vivo. Moreover, our results support the development of a multiepitope vaccine that could trigger a pan-serotype anti-dengue CD8 response.

## 1. Introduction

Dengue virus (DENV) (species: *Orthoflavivirus denguei*; genus: *Orthoflavivirus*; family: *Flaviviridae*) is an enveloped virus with a positive-sense single-stranded RNA genome. It remains a persistent global health threat, imposing a substantial burden on healthcare systems and economies worldwide. Dengue disease arises from infection with any of the four serologically and genetically distinct serotypes of the virus (DENV-1 to DENV-4). DENV is one of the most prevalent mosquito-borne arboviruses infecting humans, primarily but not exclusively transmitted by the *Aedes aegypti* mosquito [1,2]. Most DENV infections are asymptomatic or induce mild symptoms of fever, headache, body aches, nausea, and rash; however, in severe cases, they may lead to fatal situations, such as dengue hemorrhagic fever (DHF) or dengue shock syndrome (DSS) [1,3].

Infected individuals develop lasting immunity to the serotype they were exposed to, alongside a transient cross-serotype immunity. Despite the initial protection this confers against the previous serotype exposure, the antibodies so generated are non-neutralizing to a subsequent infection with a different DENV serotype. Instead, these antibodies may inadvertently enhance viral entry and promote viral replication within immune cells, a phenomenon known as antibody-dependent enhancement (ADE). ADE may lead to an exaggerated immune response and an overproduction of inflammatory cytokines, contributing to the severity of the disease [4]. Over half of the world’s population is at risk of DENV infection and its prevalence is expected to grow, driven by the forces of climate change and urbanization [5,6]. The dengue burden, with an estimated 100 million annual symptomatic cases, underscores the critical need for preventive measures [7]. Vector control measures have shown some efficacy in limiting dengue transmission. However, to optimize their efficiency, integration with a therapeutic approach is essential. The current lack of a specific antiviral therapy has prompted the emphasis on a combinatorial intervention approach, involving vaccination and vector control strategies [8,9,10].

Vaccination against DENV has demonstrated potential in providing protection against disease and reducing the risk of severe outcomes. However, the complex nature of dengue–immunity, characterized by the phenomenon of antibody-dependent enhancement (ADE) induced by the priming serotype against the others, presents formidable challenges in vaccine development [11,12]. Indeed, the ideal vaccine needs to protect against all serotypes without inducing ADE. However, existing viral vectored vaccines have exhibited variable efficacy across different serotypes and populations, often influenced by host factors such as serostatus and age. For instance, a current live-attenuated vaccine—Chimeric Yellow fever virus–DENV recombinant Tetravalent Dengue Vaccine (CYD-TDV), commercially marketed as Dengvaxia^®^ (Sanofi Pasteur Inc., Swiftwater, PA, USA)—elicits a humoral response that heightens the risk of severe dengue and hospitalization in individuals without previous exposure to dengue [13]. It is, thus, not recommended in endemic regions for children under six years old or in unexposed population [14]. Moreover, the efficacy of the Dengvaxia^®^ vaccine varies against each serotype: higher against DENV3 and DENV4 compared to DENV1 and DENV2 serotypes [15]. The other live-attenuated vaccines, TAK-003 (Takeda Pharmaceuticals, Cambridge, MA, USA) and TV003 (U.S. National Institutes of Health, Bethesda, MD, USA), currently in trial phase, each show a discrepancy in both immunogenicity and clinical efficacy against different DENV serotypes [16,17]. Awaiting further data on the effectiveness of these vaccines in phase III trials, there is a concern that individuals lacking prior exposure to the virus (seronegative) may face an elevated risk of severe dengue due to ADE associated with these vaccines. Additionally, in regions marked by the prevalence of Zika virus (ZIKV), another flavivirus sharing structural and genetic features with DENV, cases of ADE have been documented in individuals initially infected with ZIKV and subsequently with DENV2 [18]. Animal studies indicate that this phenomenon occurs with both DENV and ZIKV when live-attenuated vaccines are administered [19]. Ensuring that vaccines protect against all dengue serotypes without inducing ADE is a major challenge.

One promising avenue for enhancing vaccine efficacy lies in harnessing cellular immunity against DENV. Host cellular immune responses capable of directly eliminating virus-infected cells, regardless of serotype, hold potential for mitigating the clinical severity of dengue [20]. This approach has driven the exploration of multiepitope-based immunogens that can induce robust and cross-reactive T-cell responses against dengue antigens [21]. Such an approach not only aims to provide broader protection but also seeks to address the challenges posed by ADE.

Several murine studies indicate the protective role of CD8^+^ T cells in controlling the DENV infection and preventing antigen-induced antibody-dependent enhancement of dengue disease [22,23,24,25]. In addition, immunoproteomics work in the past years has led to the identification of novel HLA class I-specific conserved epitopes from DENV-infected cells that induce a cross-reactive cytotoxic T-lymphocytes (CTLs) response across all serotypes [26]. Various studies have utilized immunoinformatic tools to design multiepitope vaccine platforms to target CD8^+^ T cells against DENV [27,28]. However, there is a lack of in vivo studies assessing the functionality of these vaccine models, specifically in terms of multivalent cellular response. In our prior research, the in silico evaluation of a multi-epitope chimera based on HLA Class I CTL epitopes displayed potential for eliciting a CD8-specific cellular immune response [29].

We, thus, designed a novel multi-epitope-based dengue-immunogen consisting of MHC-I binding CD8^+^ T cell epitopes and we tested its capacity to induce cellular response against four DENV serotypes. The rationale behind our multi-epitope design is rooted in the idea that simultaneously targeting diverse epitopes across all serotypes may augment the vaccine’s effectiveness, inducing a broader and more robust multivalent immune response. This approach enhances protection by overcoming antigenic variation, promoting a lasting immune memory, and customizing the vaccine for specific populations. Given that the envelope (E) protein of DENV plays a pivotal role in infectivity, we identified the most promising MHC class I-binding epitopes from the E protein. The chimera consists of five epitopes per serotype, derived from conserved regions of the DENV E protein, that are predicted to bind the mouse MHC associated with the H-2^d^ haplotype. Utilizing bioinformatics tools, we designed the nucleotide sequence encoding the immunogen, merging a total of twenty epitopes with spacer peptides. Subsequent in silico assessments evaluated the structural stability and immunogenicity of the construct. In this investigation, we further examined its effectiveness in CB6F1 mice, an F1 hybrid strain of BALB/c and C57BL/6, which display a more equilibrated T cell response than BALB/c mice [30].

We showed that CB6F1 mice were able to mount a CD8 T cell response specific to all four DENV serotypes following immunization with bone-marrow-derived dendritic cells (BMDCs) expressing the multiepitope-based immunogen. Our study contributes to the ongoing pursuit of effective dengue vaccines capable of conferring cross-serotype protection and thereby alleviating the global health burden posed by DENV infection.

## 2. Materials and Methods

### 2.1. Study Design

In the study, we have experimentally evaluated a polyepitope chimeric immunogen aimed at eliciting a T cell response against DENV peptides. We designed the nucleotide sequence encoding the immunogen using immunoinformatic and proteomics tools, where we carried out the structural stability and immunogenicity analyses of the chimera in silico. Further, CB6F1 mice were used as animal model to assess the cellular immune response generated by our immunogen.

### 2.2. Mice

Six-weeks-old CB6F1 female mice were purchased from Charles River Laboratories (L’Arbresle, France) and were housed under SPF conditions in our animal facility (AniRA-PBES, Lyon, France). The research project was approved by a local ethics committee (CECCAPP, registered as CEEA015 by the French Ministry of Research) and subsequently authorized by the French Ministry of Research. All procedures were in accordance with the European Community Council Directives of 22 September 2010 (2010/63/EU) regarding the protection of animals used for scientific purposes.

### 2.3. Cell Line

Human Embryonic Kidney (HEK) 293 cell line was obtained from the American Type Culture Collection (ATCC). These adherent cells were cultured in Dulbecco’s modified Eagle medium (DMEM; Gibco, New York, NY, USA) supplemented with 10% fetal bovine serum (FBS) at 37 °C in 5% CO_2_ atmosphere.

### 2.4. DENV Peptides

The MHC-I binding epitope peptides, each having 9 amino acids in length, were commercially manufactured in lyophilised form under strict high-level quality control processes of ISO 9001 certified, Total Quality Management (TQM) platform of GenScript, USA. All the epitope peptides used in this study are presented in Table 1. The purity (>95%) and identity of peptides were ensured by mass spectrometry (MS) and reverse-phase high performance liquid chromatography (RP-HPLC) analyses. Peptides were dissolved at 20 mM in ultrapure water, dimethyl sulfoxide, formic acid, N-methyl-2-pyrrolidone or 3% ammonia water, as per manufacturer’s recommendation. The five peptides from each serotype were combined into four distinct sets (DENV1, DENV2, DENV3, DENV4) and another set containing all twenty peptides, denoted as Pool20, each at a concentration of 10 µM. For experiments, all peptides were utilized at a concentration of 100 nM to ensure optimal CD8 T cell activation.

### 2.5. Proteome Retrieval for Antigen Prediction and MHC-I T Cell Epitope Prediction

The proteomics and immunoinformatics study of the immunogen was performed, as described previously [29]. Briefly, the amino acid sequences of the envelope (E) protein of each DENV serotype group were retrieved from the Virus Variation–Dengue Virus Database hosted by the National Center for Biotechnology Information (NCBI) (https://www.ncbi.nlm.nih.gov/genome/viruses/variation/, accessed on 3 December 2021) [31]. Multiple sequence alignment (MSA) of each group was performed using the Clustal Omega algorithm, and conserved domains were identified using BioEdit software (v 7.1), with a parameter of minimum segment length of 15 amino acids.

The MHC-I binding cytotoxic T lymphocyte (CTL) epitopes were predicted using the NetMHCpan (v 4.1) tool available on the Immune Epitope Database and Analysis Resource (IEDB) (http://tools.iedb.org/mhci/, accessed on 3 December 2021) [32]. However, all epitopes of 9-mer length were predicted for mouse MHC Class I alleles and ‘d’ haplotype (H-2-D^d^, H-2-K^d^, H-2-L^d^). Following a combined prediction for proteasomal processing, TAP transport, and MHC-I binding, epitopes presented by MHC-I molecules were tallied, using IEDB T cell epitope-processing prediction tool (http://tools.iedb.org/mhci/, accessed on 3 December 2021) [33].

### 2.6. Design of the Nucleotide Sequence Encoding the Immunogen and Its Subcloning in Expression Vector

**Figure 1:**
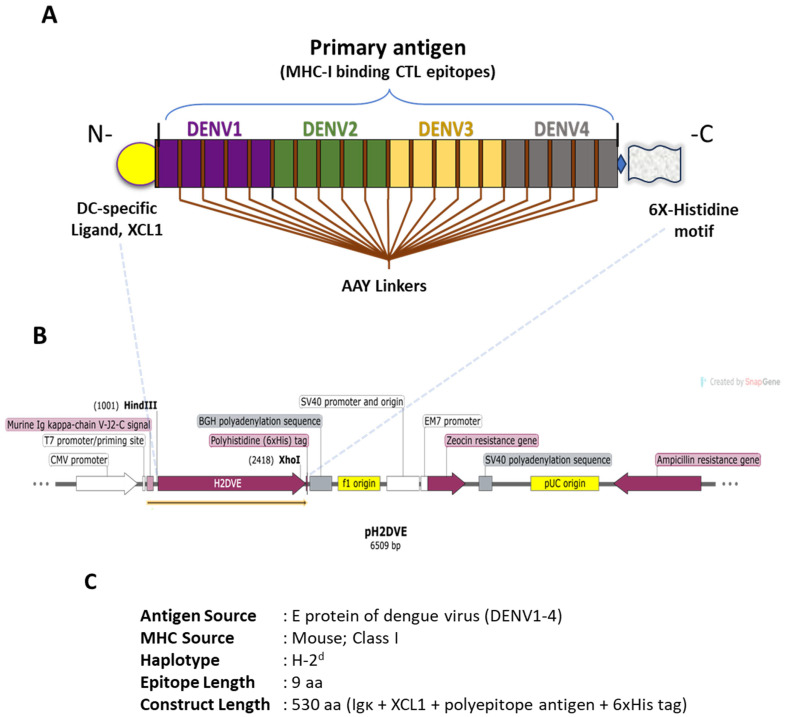
Schematic representation of the immunogen design and expression cassette subcloned in the pSecTag2B plasmid (referred to as ‘pH2DVE’ in the text). (**A**) The recombinant immunogen comprises of a dendritic cell-specific XCL1 ligand (yellow) at the N-terminus, merged with flexible and rigid linkers to sub-cassettes of MHC Class I binding epitopes from the DENV E protein (the primary antigen of the construct). The selected epitopes specific to the four dengue serotypes are represented by colored boxes (DENV1 in purple, DENV2 in green, DENV3 in light yellow, and DENV4 in grey), each connected by flanking AAY linker/spacer peptides. The pH2DVE cassette was designed using SnapGene Viewer software (v7.0). (**B**) The horizontal vector map illustrates the positioning of the primary antigen at the HindIII and XhoI restriction sites within the pH2DVE expression plasmid. (Note: H2DVE includes Ig-kappa light chain, XCL1, polyepitope antigen and 6XHis tag) (**C**) Description of the antigen source, MHC subclass and haplotype, length of each epitope, and the entire construct (aa: amino acids) is presented. Notably, the construct length mentioned here includes the entire open reading frame (ORF) from pH2DVE.

The top percentile ranked epitopes (five epitopes for each serotype), present in the conserved E domains, were sequentially merged using AAY spacer peptides in between each epitope (Figure 1). AAY spacer peptides have been shown to enhance epitope recognition and processing by the immunoproteasome [34,35]. To enhance flexibility for subcloning or potential modifications, two additional epitopes were incorporated at the N-terminal of the polyepitope using AAY spacers. Furthermore, a DC-specific ligand, XCL1, was also introduced at the N-terminal. The corresponding amino acid sequence was reverse-translated into a nucleotide sequence and codon-optimized for mammalian expression. Subsequently, the optimized sequence was subcloned into the pSecTag2B mammalian expression vector (Invitrogen, Carlsbad, CA, USA) by ligating its 5′-end and 3′-end at the HindIII and XhoI restriction sites, respectively (Figure S4A). The pSecTag2B vector facilitates secretory overexpression of recombinant proteins in mammalian cells, incorporating the immunoglobulin kappa (Ig-κ) light chain signal peptide at the N-terminal for efficient protein export. Additionally, it features a c-myc epitope followed by a six-tandem histidine (6xHis) tag at the C-terminus, allowing for easy purification and detection of expressed proteins. The resulting plasmid is now designated as pH2DVE, and the encoded sequence is denoted as H2DVE (H2 for mouse MHC-I and DVE for Dengue Virus Envelope protein). Similarly, for bacterial expression test, codon-optimized sequence was subcloned in the pET303/CT-His vector (Invitrogen, USA), with its 5′-end and 3′-end ligated at the XbaI and XhoI restriction sites, respectively (Figure S5, pET303/CT-His/H2DVE). The synthesis of both the codon-optimized nucleotide sequences encoding the antigen was carried out by GeneArt Gene Synthesis services (Thermofisher Scientific, Carlsbad, CA, USA). The physicochemical characterization of H2DVE was performed using in silico webtools, as previously described [29].

The plasmids were propagated by transforming chemically competent *Escherichia coli* cells (DH5 alpha strain) and purified using either PureYield Plasmid Miniprep System (Promega Corporation, Madison, WI, USA) or NucleoSpin Plasmid, Mini kit (Macherey-Nagel, Düren, Germany). The correct orientation and in-frame cloning of the plasmid construct were confirmed either by whole plasmid sequencing (Plasmidsaurus, Eugene, OR, USA) or by conventional Sanger sequencing at T7 promoter/priming site and BGH-reverse priming site, along with a double restriction digestion method.

### 2.7. Production of Recombinant H2DVE Polypeptide in Mammalian Expression System

HEK293 cells, seeded at 0.5 × 10^6^ cells per well in a 6-well cell-culture grade plate (Nunc, Thermo Scientific, Waltham, MA, USA), were transfected with pH2DVE (2.5 µg/well) using Lipofectamine 3000 (Invitrogen, Carlsbad, CA, USA) as per manufacturer’s protocol using serum-free OptiMEM (Gibco) culture medium. After 4 h post-transfection, the medium was gently replaced with complete growth medium. The pcDNA3-EGFP (Addgene plasmid #13031) was used as transfection control. Additionally, pSecTag2/PSA (Invitrogen, USA), which expresses prostate-specific antigen (PSA) fused with the polyhistidine tag, and pSecTag2B (Invitrogen, USA) were employed as positive and negative expression controls, respectively.

The cells were trypsinized and washed with phosphate-buffered saline (PBS), lysed using RIPA lysis buffer (Cell Signaling Technology, Danvers, MA, USA) containing 1X Halt™ protease inhibitor cocktail, EDTA-free (Thermo Scientific), and further either sonicated or thermally lysed at 95 °C for 5 min. Cellular debris were removed by centrifugation (15,000× *g*, 15 min, 4 °C) and supernatant was collected for 12% SDS-PAGE. Following electrophoresis, the proteins were transferred to 0.2 µm PVDF membrane (MDI, Haryana, India) using a semi-dry transfer method. The membranes were blocked overnight at 4 °C in blocking buffer (PBS containing 5% bovine serum albumin (BSA) and 0.1% Tween 20), followed by overnight incubation with an anti-6X His tag monoclonal antibody [HIS.H8] (ab18184, Abcam, Cambridge, UK) at a dilution of 1:5000. After three washes in PBS with 0.1% Tween 20, the membrane was incubated with HRP-conjugated goat anti-mouse IgG H&L (ab205719, Abcam) at 1:10,000 dilution for 1 h at room temperature. Following secondary antibody incubation, the membranes were washed three times. The Clarity Western ECL Substrate (Bio-Rad, Hercules, CA, USA) was used for signal development following the kit manufacturer’s recommendations and visualized on ChemiDoc imaging system (Bio-Rad, USA). The expression and production were assessed on either whole cell lysate or on purified product using Ni-NTA immobilized-metal affinity chromatography (IMAC) spin column (Qiagen, Hilden, Germany), under denaturing conditions. Protein quantification was performed using Pierce BCA protein assay (Thermo Scientific, USA).

### 2.8. Generation of Murine pH2DVE-Transfected BMDCs

The bone marrow cells were isolated from CB6F1 mice by flushing femurs with complete DMEM (DMEM medium [Gibco] supplemented with 6% fetal calf serum [FCS] [Life Technologies, Carlsbad, CA, USA], 1% HEPES [Gibco], 1% non-essential amino acids [Gibco], 0.1% gentamycin [Gibco] and 0.1% β-mercaptoethanol [Gibco]). The cells (2 × 10^6^ cells per mL) were cultured in a complete DMEM medium supplemented with human Flt3-L (100 ng/mL) for 7 days at 37 °C in a humidified incubator containing 7% CO_2_. On day 8, BMDCs were electroporated with pH2DVE (2 µg) either in a nucleocuvette strip (5 × 10^4^ or 2 × 10^5^ cells) or in a single nucleocuvette (1.25 × 10^6^ cells), using P4 Primary Cell 4D- Nucleofector™ X Kit and 4D-Nucleofector™ unit (Lonza, Singapore), as per manufacturer’s instruction (DK-100 program). The expression of H2DVE recombinant protein by BMDCs was assessed by automated capillary-based protein separation and immunodetection on Jess ProteinSimple platform, as per manufacturer’s recommendations (primary antibody: anti-6X His tag monoclonal antibody [HIS.H8] [ab18184, Abcam] at 1:20 and secondary antibody: anti-mouse HRP-conjugated at 1:20) 6 or 24 h post-transfection.

### 2.9. Immunization

CB6F1 mice were immunized by two intravenous injections of pH2DVE-transfected BMDCs (5 × 10^4^ cells in 200 µL of PBS) by retro-orbital route, following brief anesthesia with isoflurane. Naive mice were used as a control.

### 2.10. T Cell Stimulation

Mice were sacrificed by cervical dislocation for tissue harvesting. Spleens were mechanically disrupted and filtered through a sterile 100-μm nylon mesh filter (BD Biosciences, San Jose, CA, USA). For ex vivo stimulation, splenocytes were incubated with peptide sets of Pool20, DENV1, DENV2, DENV3 or DENV4 (100 nM of each individual peptide in complete DMEM) at 37 °C in a humidified incubator containing 5% CO_2_. After 2 h, GolgiStop (BD Biosciences, USA) at 1/1000 dilution was added to block cytokine secretion, and incubated for another 4 h.

For in vitro expansion, splenocytes were stimulated with Pool20 (100 nM of each individual peptide) in the presence of IL-2 (11.5 ng/mL) and incubated for 8 days at 37 °C in a humidified incubator containing 7% CO_2_. Half of the medium was replenished with fresh culture medium containing IL-2 every third day. Expanded cells were then restimulated, as described above, for ex vivo stimulation.

### 2.11. Enzyme Linked Immunosorbent Assay (ELISA)

The interferon gamma (IFNγ) in the supernatant was quantified using ELISA MAX™ Standard Set for mouse IFNγ (BioLegend, San Diego, CA, USA) following manufacturer’s instructions. Absorbance at 450 nm was measured using an Infinite 200 microplate reader (TECAN, Männedorf, Switzerland).

### 2.12. Flow Cytometry

Cells were first stained with efluor780-coupled Fixable Viability Dye (Thermo Scientific) for 15 min at 4 °C and then incubated with an Fc receptor blocking antibody (2.4G2 hybridoma supernatant) for 10 min at 4 °C. Surface staining was performed for 30 min at 4 °C with the appropriate mixture of antibody (Ab) diluted in staining buffer (PBS supplemented with 1% FCS [Life Technologies] and 0.09% NaN_3_ [Sigma-Aldrich, St. Louis, MO, USA]). Cells were then fixed and permeabilized using CytoFix/CytoPerm buffer according to manufacturer’s instructions (BD Biosciences) and stained with the intracellular Ab-cocktail for 30 min at 4 °C. Antibodies used are listed in the Supplementary Table S3. All analyses were performed on a BD LSRFortessa cell analyzer (BD Biosciences) and further analyzed using FlowJo™ v9.3 software (BD Life Sciences, Franklin Lakes, NJ, USA).

### 2.13. Statistical Analyses

Data were expressed as arithmetic median ± range. Mann–Whitney tests (comparison of two groups) were used to compare unpaired values (GraphPad Prism v10). Significance is represented: * *p* < 0.05.

## 3. Results

### 3.1. Identification of T Cell Epitopes from DENV E Protein

The envelope (E) protein sequences of each serotype were retrieved using the search criteria described in Methods from the Virus Variation–Dengue Virus Database. There were 1530 sequences for the DENV1 serotype, 1022 for DENV2, 448 for DENV3 and 304 for DENV4. The conserved domains were identified for each serotype after the multiple-sequence alignment relative to the corresponding reference sequences (Table S1).

The IEDB prediction method NetMHCpan EL 4.1 predicted different peptides for each serotype using the distinct reference sequence of the E protein. We sorted the epitope selection with an integrated and stepwise approach for downstream prediction analyses. In the first step, epitopes with PR ≤ 1.5 in either H-2D^d^ or H-2K^d^ specific MHC class-I binding prediction, which were present in the conserved domains of the E protein of the respective serotype, were shortlisted (Table S2). Further, a combined prediction was performed for proteasomal processing, TAP transport, and MHC-I binding using IEDB T cell epitope-processing prediction tool. For each serotype, five key epitopes showing the intrinsic potential of a T cell epitope were selected for the multivalent immunogen construct (Table 1).

### 3.2. Immunogen Construction and Its Physicochemical Characterization

**Figure 2:**
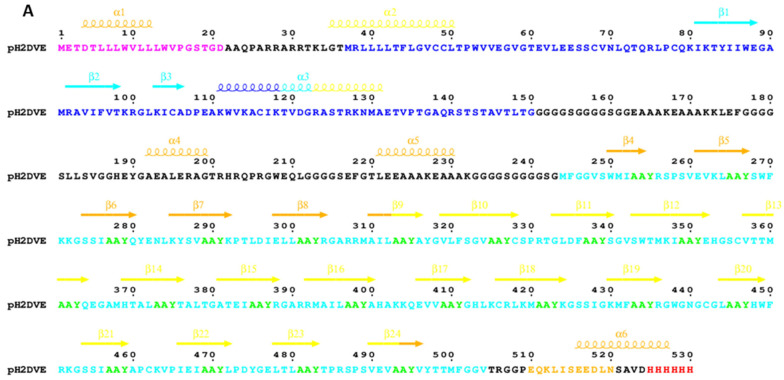

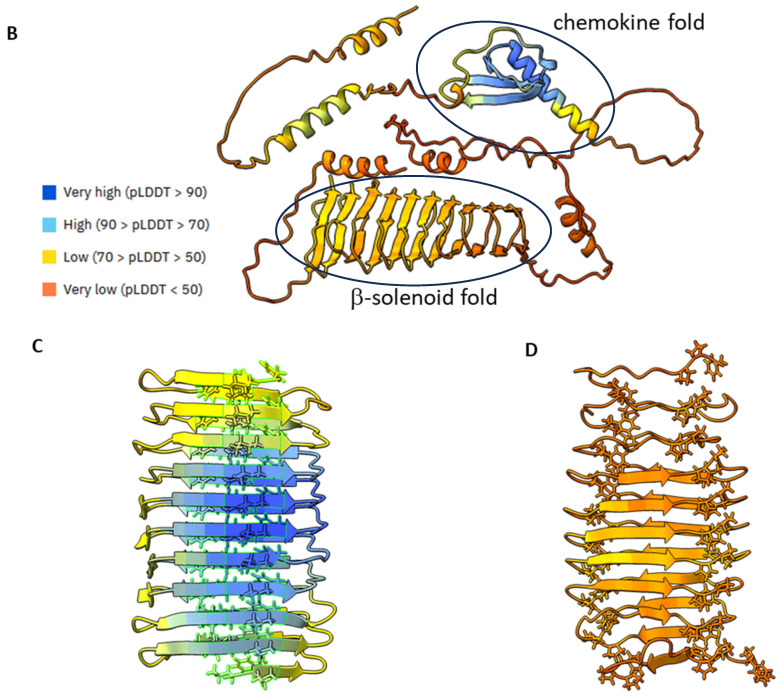
(**A**) The produced H2DVE sequence that comprises the Ig-κ signal peptide (purple), the XCL1 sequence (blue), the polyepitope sequence (cyan) with AAY spacers (green), a c-myc epitope (orange), and a polyhistidine tag (red); it is adorned with secondary structure elements extracted from the AlphaFold model and colored according to the pLDDT confidence score (blue for very high, cyan for high, yellow for low, orange for very low). Figure prepared with ESPript [42]. (**B**) The Cα trace of the Alphafold model of H2DVE with the three-stranded β-sheet/α-helix chemokine domain of XCL1 and the β-solenoid polyepitope domain. (**C**) The high confidence AlphaFold model of the polyepitope domain with IIF spacers displayed in atom mode. (**D**) The very low confidence model of the polyepitope domain with PPF spacers. All structures are rendered with ChimeraX [43]. The blue, cyan, yellow and orange color information in (**B**–**D**) represents pLDDT confidence score as depicted within the figure.

The full-length multi-epitope sequence of the tetravalent immunogen against DENV was constructed by merging the selected nonameric amino-acid peptides from DENV1, DENV2, DENV3, and DENV4 in sequential order, incorporating a three-residue spacer (AAY) between each epitope (Figure 1 and Figure 2A). To enhance specificity for cross-presenting dendritic cells (DCs) during recombinant protein immunization, a DC-specific ligand, XCL1, was introduced at the N-terminal of H2DVE [36]. Additionally, we incorporated two distinct random epitopes specific to the DENV4 serotype (absent in conserved domains) at the N-terminal of the polyepitope (refer to Table S2 for their prediction scores). The first epitope, ‘MFGGVSWMI’, retained a start codon for potential sub-cloning into diverse plasmid vectors. The second epitope, ‘RSPSVEVKL’, was selected as a buffer for potential modifications. By incorporating a buffer epitope, we have created a designated space that can accommodate changes or additions to the polyepitope sequence without disrupting the overall structure of the primary antigen. This approach allows for the flexibility needed for future customization. Both epitopes were arranged in the epitope-spacer-epitope pattern, optimizing recognition by the proteasome. Notably, XCL1 and the polyepitope domain in H2DVE were separated by an extended spacer (Figure 2A,B) to prevent any interference with binding to DCs.

The physicochemical properties of the recombinant protein were evaluated in silico, using the tools previously described [29]. Protein-Sol webserver was used to predict the solubility of the recombinant H2DVE across its three segments: the primary antigen, XCL1 fused with the primary antigen, and the whole H2DVE sequence (including Ig-κ chain, c-myc epitope and 6xHis tag). The predicted scaled solubility (PSS) for these segments was 0.612, 0.454, and 0.395, respectively (Figure S1A). The scaled solubility value (QuerySol) represents the predicted solubility of a protein. The average solubility (PopAvrSol) is 0.45 and a QuerySol value of a protein above 0.45 indicates higher solubility compared to the average *E. coli* protein in the dataset, while values below 0.45 suggest lower solubility [37].

The tabulated results highlight crucial physicochemical properties of the construct (Table 2). The hydropathicity and transmembrane tendencies, predicted by Expasy ProtScale, indicated the hydrophilic nature of the recombinant protein with no membrane-spanning regions (Figure S2A,B). Likewise, the DeepTMHMM server (version 1.0) identified the first 21 residues in the entire amino acid sequence as a ‘signal peptide’, consistent with UniProt database annotations for the amino-terminal sequence of mouse XCL1 [38]. The remaining residues are labelled as ‘outside’, suggesting the absence of transmembrane topology, as illustrated in Figure S2C. Furthermore, the DeepCoil tool showed no significant coiled-coil domains in the protein sequence (Figure S2D).

In addition, to further validate the structural features of H2DVE, we used AlphaFold, a revolutionary artificial intelligence program that predicts three-dimensional structures of proteins [39]. The modelled structure has a per-residue confidence score (pLDDT) of 52% and shows two structured domains separated by disordered regions (Figure 2B). The first domain adopts the chemokine fold observed in the homologous known structure of human XCL1 while the second domain displays a β-solenoid fold due to the repetition of the AAY spacer along the polyepitope sequence [40,41]. The protein structures were visualized and depicted using ESPript (v3.0) and ChimeraX (v1.6) [42,43].

Furthermore, the results retrieved from AllerTop v.2.0 and AllergenFP v.1.0 servers characterized H2DVE protein as a non-allergen, suggesting that, when used as a subunit immunogen, it may not induce autoimmune or allergic reactions. The probability of H2DVE antigenicity was predicted at 0.48 by Vaxijen v2.0 and 0.54 by ANTIGENpro, indicating its potential of eliciting efficient immune responses.

### 3.3. Codon-Optimized Synthesis, Subcloning, and Expression Profiling of H2DVE Encoding Nucleotide Sequence

Using GeneOptimizer tool, negative cis-acting sites (such as splice sites, TATA-boxes, etc.), which may have negatively influenced expression, were eliminated. GC content was adjusted to prolong mRNA half-life. Codon usage was adapted to the bias of *Homo sapiens* resulting in a codon adaptation index value of 0.87, with average GC content of 57% (Figure S3). The optimized gene sequence of H2DVE was synthesized commercially using GeneArt gene synthesis services from Invitrogen and subcloned into pSecTag2B vector (Figure S4A,B). Verification of the correct orientation and desired reading frame of the final plasmid vector product (pH2DVE) was confirmed through sequencing analysis and double restriction digestion (Figure S4C).

**Figure 3:**
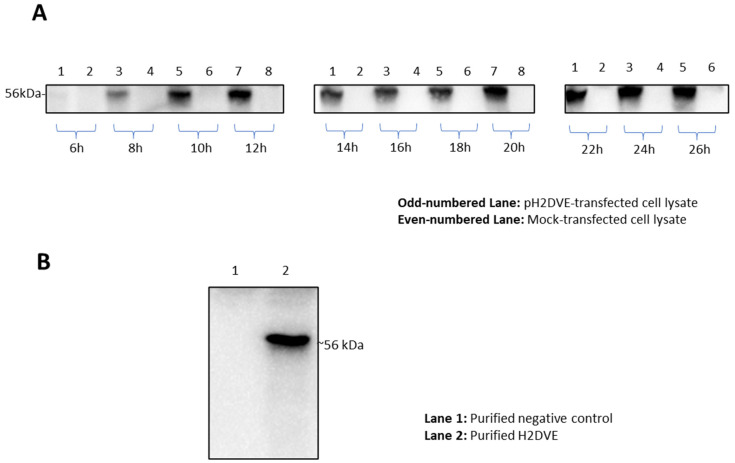
Western blots depicting the expression of H2DVE recombinant protein in a mammalian system (HEK293 cell line). (**A**) Time course expression profile using unpurified whole lysate (10^6^ cells) of pH2DVE transfected cells (odd numbered lanes) and mock-transfected (pSecTag2B, empty vector control) cells (even numbered lanes). The blot images were aligned with the 56 kDa bands for clarity and consistency. (**B**) Purified proteins from the whole lysate of mock-transfected control (lane 1) and pH2DVE transfected cells (lane 2). The recombinant proteins were tagged with poly-histidine tag at the C-terminal and were purified using Ni-NTA IMAC spin columns. All the blots were imaged using ChemiDoc system (Bio-Rad) and ImageLab software (v5) with automatic image exposure.

The resulting protein derived from pH2DVE contained the murine Igκ chain leader sequence at its N-terminus to facilitate protein secretion. The time–course expression analysis, performed on the entire lysate of HEK293 cell line transfected with pH2DVE, revealed detectable expression of the recombinant protein starting from 8 h post-transfection (Figure 3A). A whole lysate obtained after 24 h post-transfection was subjected to Ni-NTA IMAC column purification, demonstrating a distinct band at the intended position (Figure 3B).

The rationale behind choosing the mammalian expression system was, firstly, to eliminate any risk of endotoxin contamination of the purified product and, secondly, to achieve a substantial yield of H2DVE using pSecTag2 vector backbone. Indeed, mice immunization requires large quantities of immunogen. Unfortunately, despite the efforts, the purification yield in the current system was insufficient (750 µg/mL), and all attempts to concentrate the protein proved to be unsuccessful. This presented a significant challenge in meeting the demands of our immunization study. Transitioning to the bacterial expression system using various *E. coli* strains exhibited low expression levels, indicating potential insolubility or undetectable expression.

### 3.4. Recombinant H2DVE Is Expressed by the Bone-Marrow Derived Dendritic Cells

**Figure 4:**
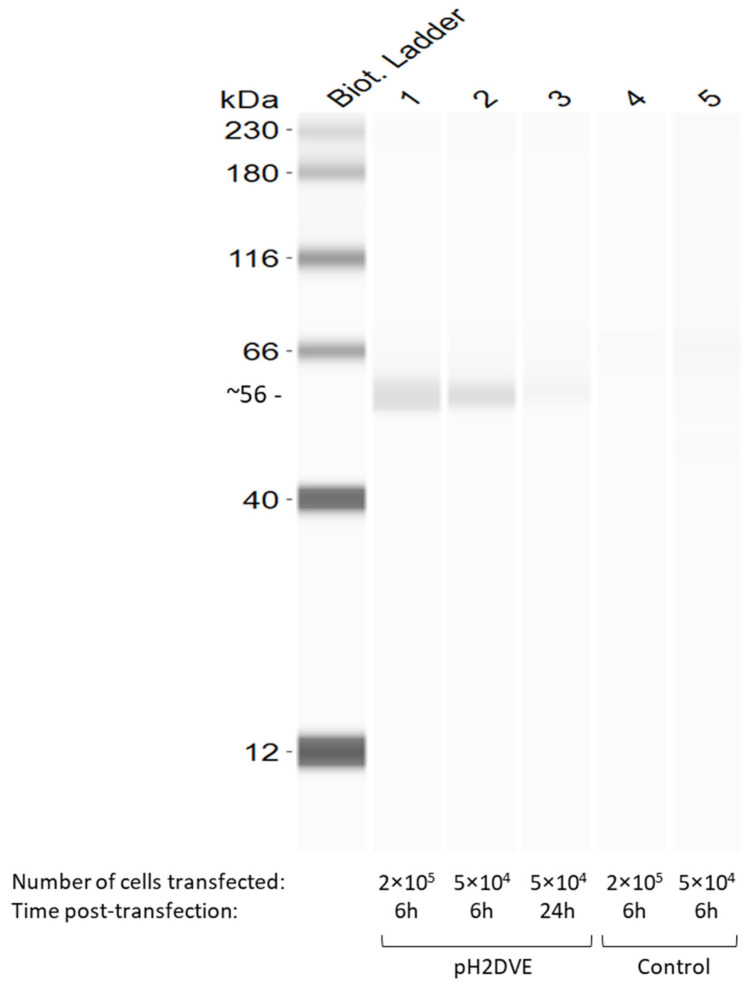
Jess SimpleWestern Immunoblot showing the H2DVE expression profile in pH2DVE transfected BMDCs (lane 1 to 3) and non-transfected control (lane 4 and 5). The immunoblot images were analyzed using ‘Compass for SimpleWestern’ software (v6.3).

Due to suboptimal yields in protein production, our approach was refined to utilize pH2DVE-transfected BMDCs from syngeneic CB6F1 mice. The expression profile of the recombinant protein in transfected BMDCs was examined at 6- or 24 h post-transfection. Notably, we observed a better H2DVE expression at the 6 h time point compared to the 24 h time point, as illustrated in Figure 4. These findings indicate that transfected BMDCs were able to express the recombinant poly-epitope construct.

### 3.5. Immunization with pH2DVE-Transfected BMDCs Primed a CD8 T Cell Immune Response to DENV Peptides

**Figure 5:**
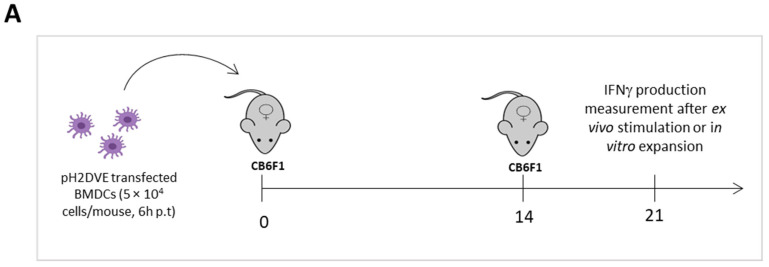

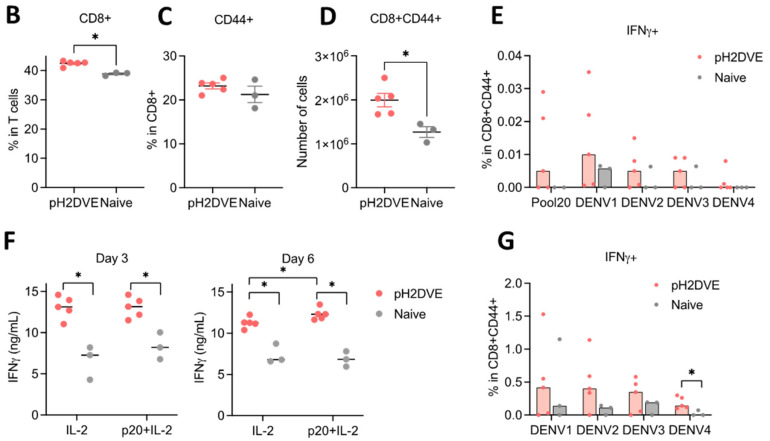
CB6F1 mice were immunized twice with pH2DVE-transfected BMDCs at a two-weeks interval and H2DVE-specific CD8 T cell response was evaluated one week after second immunization and compared to naive mice (**A**). The percentage of total CD8^+^ (**B**) and CD44^+^ cells within CD8 T cells (**C**) were measured by flow cytometry. The number of CD44^+^ CD8^+^ T cells were determined (**D**). Total splenocytes were stimulated with DENV specific peptide pools Pool20, DENV1, DENV2, DENV3 and DENV4 (100 nM) for 6 hours and the expression of IFNγ was measured by flow cytometry (**E**). Total splenocytes were expanded in the presence of Pool20 peptides (100 nM) and IL-2 (11 ng/mL) or IL-2 alone for 8 days. The production of IFNγ was followed over time in the supernatants by ELISA (**F**). Following expansion, cells were restimulated as described in (**E**) and IFNγ production was measured by flow cytometry (**G**). One represented experiment out of two (N = 3–5/group). The statistical significance of differences was determined by Mann-Whitney test (* *p* < 0.05).

To address the immunogenicity of H2DVE, CB6F1 mice received two injections of pH2DVE-transfected BMDCs at a two-weeks interval, and antigen-specific CD8 T cell response was evaluated one week after the second immunization (Figure 5A). We observed an increased proportion of CD8^+^ T cells within the T cell pool (Figure 5B), as well as an increased proportion of CD44^+^ cells among the CD8^+^ T cell (Figure 5C) associated with an increased number of CD8^+^CD44^+^ cells (Figure 5D) in immunized mice as compared to naive mice.

Splenocytes were restimulated with a pool of peptides corresponding to the 20 peptides (Pool20) encoded in the H2DVE recombinant protein, or with a 5-peptide pool corresponding to the DENV1, DENV2, DENV3 or DENV4 derived peptides (100 nM) for 6 h and, the ability of CD8 T cells to respond to each serotype was evaluated by flow cytometry. The CD8 T cells were able to produce IFNγ in response to all peptide pools except the DENV4-pool in 3 out of 5 mice (Figure 5E). We observed a non-specific response in naive mice following stimulation with DENV1 pool peptides, but not with other peptide pools.

As the proportion of IFNγ-expressing CD8 T cells was extremely low, antigen-specific CD8 T cells were expanded in response to stimulation with the Pool20 (100 nM) in the presence of IL2 (11.5 ng/mL). Higher levels of IFNγ were detected in the supernatant of expanded cells from H2DVE-immunized mice after 3 or 6 days of expansion compared to those from naive mice, confirming that CD8 T cells were activated by priming with H2DVE (Figure 5F). Following 6 days of expansion, cells expanded with Pool20 + IL-2 exhibited slightly higher IFNγ production compared to those expanded with IL-2 alone (Figure 5F). Subsequently, after 8 days of expansion, cells were restimulated with the different peptide pools for 6 h and the production of IFNγ was measured by flow cytometry. We observed an increased proportion of IFNγ-producing CD8 T cells in H2DVE vaccinated mice following expansion with all peptide pools (Figure 5G).

Altogether, our results demonstrate that H2DVE effectively triggered a CD8-specific response against the MHC-I epitopes derived from DENV E protein across all serotypes.

## 4. Discussion

Our study introduces a novel multi-epitope-based antigen, H2DVE, designed to induce a CD8^+^ T cell response against MHC-I epitopes derived from the DENV E protein across all serotypes. Long-term immunity is induced following infection with one DENV serotype, but subsequent cross-serotype infections generate only a short-term or incomplete heterotypic protection. Complications arise from non-neutralizing antibodies, which enhance virus particle uptake by Fcγ-receptor-bearing cells, leading to severe manifestations like DHF/DSS.

The development of an effective multivalent vaccine against DENV has been a challenging endeavor due to the risk of ADE [16]. We selected the structural protein (E protein) as the CTL epitope source of our vaccine design because of its antigenic abundance in native viral infection [44]. Recent recombinant preclinical vaccines that are designed to elicit a humoral response have shown poor immunogenicity for the DENV E protein [21,45,46]. Additionally, studies indicate variability in CD8^+^ T cell responses among different serotypes, with responses tuned to non-structural proteins for serotype 1, 2, and 4, and to both structural and non-structural proteins for serotype 3 [21]. However, these studies predominantly investigated HLA transgenic mice and may not be accurately representative of immunodominant viral proteins.

During proteome retrieval of DENV E protein, from the Virus Variation–Dengue Virus database, the presence of hundreds of variant sequences for different DENV serotypes highlights the genetic diversity within each serotype and the need for a multivalent vaccine strategy. The most promising T cell epitopes, identified within conserved regions of the DENV E protein and specific to H-2D^d^ or H-2K^d^, were selected based on their binding affinity to MHC-I alleles. Specifically, the ‘d’ haplotype was used in both prediction and immunogen design, given the previous selection of BALB/c mice. However, the CB6F1 mouse, an F1-hybrid of female BALB/cAnNCrl and male C57BL/6NCrl strains, was chosen to address the Th2 or Th1 bias associated with these strains, respectively [30]. Further, the use of integrated prediction tools for proteasomal processing, TAP transport, and MHC-I binding ensured that the selected epitopes had substantial potential to elicit a robust T cell response [29].

Immunization of mice requires large quantities of subunit antigens to induce noticeable immune responses [47,48]. However, when we expressed H2DVE in mammalian cells, despite producing detectible levels after 8 h, the yield was not sufficient for purification of large quantities of protein. Nevertheless, effective T cell priming remained the key objective of the study. Consequently, we altered our approach, opting to utilize pH2DVE-transfected BMDCs from syngeneic CB6F1 mice for antigen presentation. Our findings illustrate that mice immunized with H2DVE were able to mount a detectable IFNγ-CD8 T cell response to the four DENV serotypes. These findings were particularly important given the central role of CD8^+^ T cells in antiviral immunity [24]. An extended study would be required to fully characterize the priming potential of pH2DVE-transfected BMDCs, especially considering that individual epitopes were not tested at this stage. However, it is first necessary to focus on a further refinement of our multi-epitope design, especially in terms of its recombinant production and stability.

Recombinant proteins are typically generated using expression systems based on high performance cell lines or genetically engineered bacteria. However, these proteins derived from synthetic nucleotide sequences may not seamlessly align with the principles of natural biochemistry. Understanding the protein’s structure is crucial, as it often leads to significant insights into its function, especially when existing knowledge is limited. In our study, we exploited AlphaFold to predict the structural properties of the protein expressed by pH2DVE. The first segment of the model that comprised XCL1 was mostly disordered, while the second segment with the polyepitope region exhibited a well-defined β-solenoid conformation due to the repeated AAY motif (Figure 2A,B). This disparity indicated suboptimal overall structural integrity in our protein design, potentially contributing to the challenges we encountered during its recombinant production, and we will shorten the spacer between XCL1 and the polyepitope region in future constructs. It can also be noted that the predicted polyepitope tertiary structure remained consistent even without the Ig-κ and XCL1 domains.

Since the polyepitope domain is critical for the cytotoxic immune response and is modelled with a β-solenoid fold dependent on the sequence of the repeating spacer, we performed new predictions with AlphaFold on this domain, adhering to the “aliphatic-aliphatic-aromatic” motif of AAY. The resulting structures had a pLDDT confidence score ranging from 0.78 for an IIF spacer to 0.40 for a PPF spacer (Figure 2C,D), with AAY scoring 0.62. As anticipated, all structures featured a β-solenoid fold, with the repeating spacers positioned one below the other in a ladder-like structure. Structures with scores exceeding 0.7 indicate both reliability and stability, and, notably, in the IIF spacer, the aromatic residue points inward, enhancing rigidity of the protein core within the β-solenoid fold (Figure 2C). Conversely, scores below 0.5 indicate less reliability and flexibility, as observed in the PPF model with shorter strands that do not include the repeat motif (Figure 2D). These structural differences may play a role in the proteolytic cleavage of H2DVE by the proteasome and will, therefore, be interesting to investigate.

Overall, the structural insights gained through AlphaFold prediction contribute to our understanding of H2DVE’s molecular architecture and may have implications for further improvements of its production and/or its immunogenicity. Further investigations are warranted to elucidate the functional significance of these structural features in the context of protein production, immune response, and vaccine design.

Despite the promising findings presented in the study, several limitations need to be considered. Firstly, the in vivo testing was relatively limited, and the number of mice used was low. Furthermore, the study lacks in vivo viral challenge and protection analysis in terms of proliferation or CTL assays, which are essential for evaluating the effectiveness of the vaccine. Unintended humoral response was not measured, and considering the potential risk of ADE, this aspect needs to be addressed in future research to ensure the safety of the vaccine candidate. Additionally, the chimeric protein, H2DVE, exhibited challenges in recombinant production, and the study acknowledges the need for further refinement in this aspect. While the study emphasizes the importance of CD8^+^ T cell response, the activation observed was low, and the efficacy of such a response needs further investigation. Furthermore, the use of serotype-specific pools for T cell activation might not capture the full diversity of responses, as individual epitopes should be studied. It may be crucial for selecting the most effective epitopes in each DENV serotype for a refined multi-epitope design. Moreover, the choice of the delivery method—whether as a recombinant subunit antigen or a nucleic acid vaccine—requires careful consideration of various factors. These factors include the physicochemical characteristics of the vaccine, the target population, logistical considerations, and safety profiles. These aspects call for further in-depth exploration to identify the most suitable and effective delivery strategy for H2DVE, thereby advancing its potential as a viable vaccine candidate.

In summary, our study presents a comprehensive approach to the design and evaluation of a multi-epitope tetravalent immunogen, H2DVE, against dengue virus. Successfully expressed in dendritic cells, H2DVE induced a specific CD8^+^ T cell response against all four DENV serotypes in a murine model. These findings hold promise for the development of a vaccine that can effectively address the challenges posed by DENV diversity and the risk of ADE. Further research and preclinical trials will be necessary to validate the potential of H2DVE as a candidate vaccine against the dengue virus.

## Figures and Tables

**Table 1:** List of top percentile MHC-I binding CTL epitopes. The enlisted epitopes with PR ≤ 1.5 (five for each serotype) below are present in conserved regions and are specific * to their respective DENV serotypes.

MHC Class I Binding T Cell Epitopes	Proteasome Score ^1^	TAP Score ^2^	MHC Score ^3^	Processing Score ^4^	Total Score ^5^	MHC Binding Score ^6^	Percentile Rank (PR) ^7^
DENV1							
SWFKKGSSI	0.88	0.43	−2.01	1.31	−0.70	0.660011	0.04
QYENLKYSV	0.91	0.21	−3.01	1.12	−1.89	0.553404	0.07
KPTLDIELL	1.59	0.25	−3.76	1.84	−1.92	0.342336	0.16
RGARRMAIL *	1.64	0.48	−2.60	2.12	−0.48	0.136102	0.09
AYGVLFSGV	0.87	0.25	−2.94	1.12	−1.82	0.383199	0.16
DENV2							
CSPRTGLDF	1.14	1.02	−3.48	2.16	−1.32	0.058325	0.3
SGVSWTMKI	1.14	0.13	−3.36	1.27	−2.09	0.035419	0.57
EHGSCVTTM	1.18	0.00	−3.42	1.17	−2.24	0.054472	1.3
QEGAMHTAL	1.44	0.32	−3.98	1.76	−2.22	0.047737	1.5
TALTGATEI	1.09	0.24	−2.95	1.33	−1.62	0.046412	1.5
DENV3							
RGARRMAIL *	1.64	0.34	−2.60	1.98	−0.62	0.136102	0.09
AHAKKQEVV	1.20	0.27	−2.98	1.47	−1.51	0.091084	0.84
GHLKCRLKM	0.87	0.13	−3.15	1.00	−2.15	0.053015	1.3
KGSSIGKMF	1.41	1.07	−4.06	2.49	−1.57	0.02693	0.78
RGWGNGCGL	1.46	0.42	−3.79	1.88	−1.91	0.01558	1.4
DENV4							
HWFRKGSSI	0.93	0.37	−2.36	1.30	−1.06	0.463186	0.11
APCKVPIEI	1.42	0.15	−3.60	1.57	−2.03	0.459134	0.09
LPDYGELTL	1.54	0.25	−3.62	1.79	−1.83	0.36346	0.14
TPRSPSVEV	1.14	0.02	−3.74	1.16	−2.58	0.263743	0.27
VYTTMFGGV	0.92	0.28	−2.78	1.20	−1.58	0.260915	0.29

^1^ Proteasome cleavage scores represent logarithmic values of the total amount of cleavage site usage liberating the peptide C-terminus. Higher values indicate a higher likelihood of proteasomal cleavage at multiple sites along the protein sequence. ^2^ TAP transport score estimates effective −log(IC50) values for the binding affinity of a peptide to TAP. TAP transport rates are proportional to the score values. ^3^ MHC score is given in −log(IC50) values; sign change is only for consistency, where higher values are associated with higher predicted efficiency. ^4^ Processing score combines the proteasome and TAP scores. It predicts a quantity proportional to the amount of peptide present in the ER, where a peptide can bind to multiple MHC molecules. This allows for the prediction of T-cell epitope candidates independent of MHC restriction. ^5^ Total score is the combined score of the proteasomal cleavage, TAP transport and MHC binding predictions. It predicts a quantity proportional to the amount of peptide presented by MHC molecules on the cell surface. ^6^ MHC binding score indicates MHC binding prediction, where high score displays good binder. It predicts the likelihood of a peptide being an MHC ligand. ^7^ Percentile rank (PR) informs if a peptide sequence is a strong binder (SB) or weak binder (WB) to MHC. PR of 1 indicates that the prediction score for the queried sequence falls within the top 1% of scores observed among randomly selected natural peptides. As a default, the thresholds for detecting SB and WB are set at PR below 0.5% and 2%, respectively. * RGARRMAIL peptide was an exception to serotype specificity, as it was associated with both DENV1 and DENV3.

**Table 2:** The physicochemical properties of H2DVE predicted by Expasy ProtParam and ProtScale, Protein-Sol, DeepTMHMM, DeepCoil, Vaxijen v2.0, ANTIGENpro, AllerTOP v. 2.0, AllergenFP v.1.0, PSIPRED.

Property	Value/Score *	Interpretation/Result
No. of amino acids	530	
Molecular Weight	56 kDA	-
Theoretical pI	9.22	Basic
Half-life (mammalian)	30 h	-
Half-life (yeast)	>20 h	-
Half-life (*E. coli*)	>10 h	-
Aliphatic Index	77.51	Thermostable
Instability Index II	34.99	Stable
Hydropathicity (GRAVY)	−0.041	Hydrophilic
Solubility (Protein-Sol)	0.554	Soluble
Solubility (SOLpro) (for primary antigen)	0.637505	Soluble
Transmembrane tendency (ProtScale and DeepTMHMM)	-	No transmembrane state
Coiled-coil structures (DeepCoil)	-	No coiled-coil domains
Allergenicity	-	Non-allergen
Antigenicity		Antigenic
Vaxijen	0.48	
ANTIGENpro	0.54	
Helices	28.95%	-
Coils	47.57%	-
Strands	23.48%	-

* Some tools indicate only the result as outcome and no score values.

## Data Availability

Data are contained within the article or Supplementary Materials.

## Supplementary Materials

The following supporting information can be downloaded at: https://www.mdpi.com/article/10.3390/vaccines12030316/s1, Figure S1: Protein-Sol solubility calculation results for the H2DVE recombinant protein; Figure S2: H2DVE in silico characterization; Figure S3: GeneOptimizer Assisted Sequence Analysis of H2DVE encoding nucleotide sequence; Figure S4: Plasmid sequence map of pH2DVE and pSecTag2/PSA, and their restriction digestion results; Figure S5: Plasmid sequence map (pET303/CT-His/H2DVE); Table S1: Conserved sequences in the envelop protein of four serotypes of dengue virus; Table S2: Selection of MHC-I binding epitope peptides present in the E protein conserved regions; Table S3: List of antibodies used for flow cytometry.
