# Supplementary material for: Unveiling a Shield of Hope: A Novel Multiepitope-Based Immunogen for Cross-Serotype Cellular Defense against Dengue Virus
Source: Vaccines (Basel). 2024 Mar 16;12(3):316. doi: 10.3390/vaccines12030316 (PMC10975250; doi:10.3390/vaccines12030316)
Supplement: Supplementary file 1 [file vaccines-12-00316-s001.zip › Supplementary Figures.pdf]

A

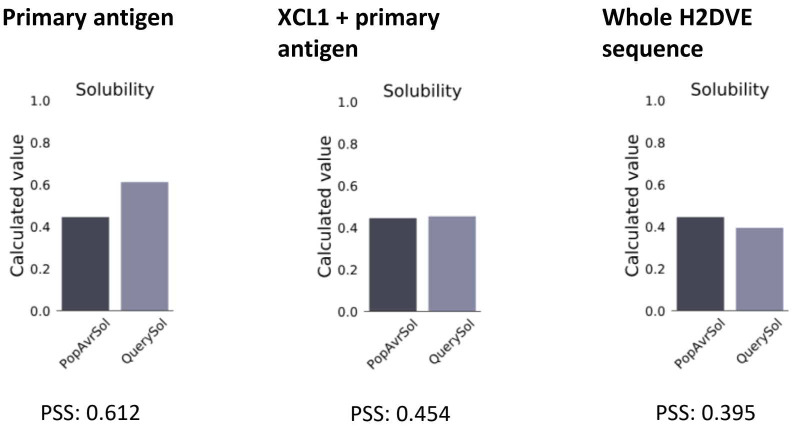

B

Polyepitope

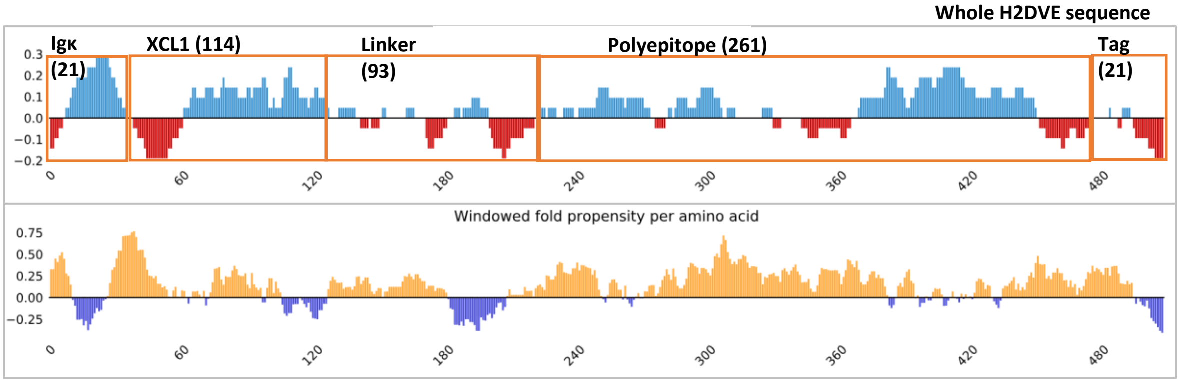

Figure S1: Protein-Sol solubility calculation results for the H2DVE recombinant protein. **(A)** PSS scores of three H2DVE segments: primary antigen alone; XCL1 (GenBank: AAA56752.1) fused with primary antigen; and the entire H2DVE sequence. **(B)** Plots depicting windowed fold propensity and windowed net charge for the whole H2DVE sequence, with residues lengths indicated in parentheses. (PSS: predicted scaled solubility)

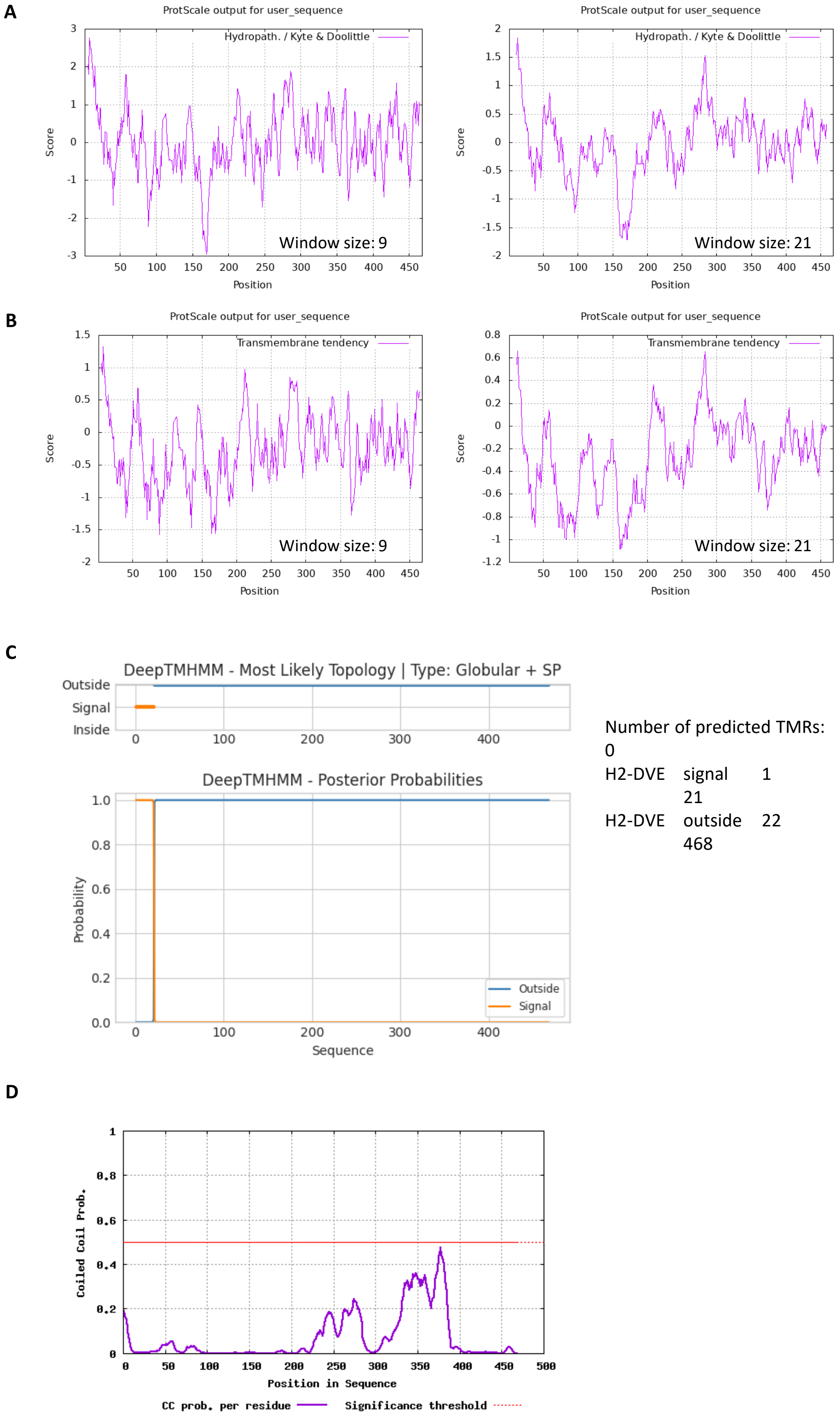

Figure S2: H2DVE characterization through various analyses: **(A)** Hydropathicity assessed using the Kyte & Doolittle method on the Expasy ProtScale server, **(B)** Transmembrane tendency evaluated with window sizes of 9 and 21, considering relative weight variations between window edges and center (100%), **(C)** Transmembrane topology prediction using DeepTMHMM (TMRs: transmembrane regions), and **(D)** Coiled-coil domain prediction.

A

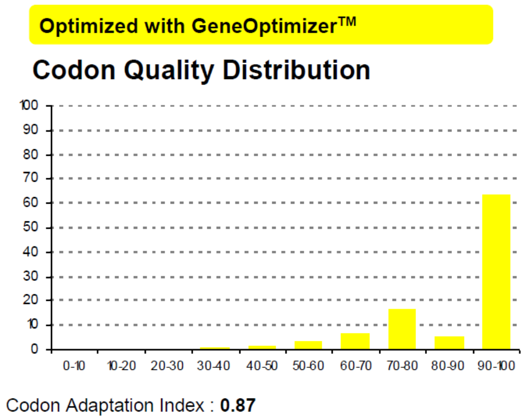

B

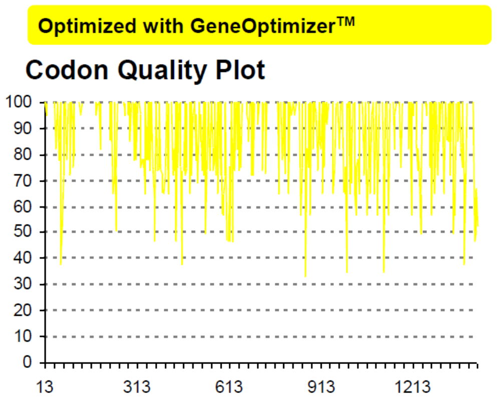

C

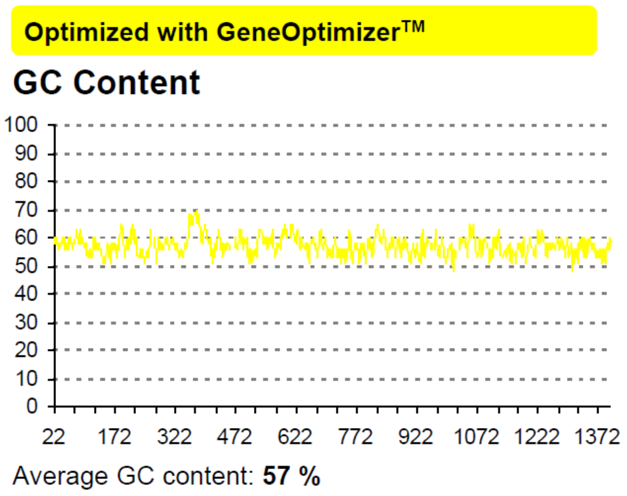

Figure S3: GeneOptimizer Assisted Sequence Analysis of H2DVE encoding nucleotide sequence. **(A)** The histograms show the percentage of sequence codons which fall into a certain quality class. The quality value of the most frequently used codon for a given amino acid in the desired expressed system is set to 100, the remaining codons are scaled accordingly. **(B)** The plots show the quality of the used codon at the indicated codon position. **(C)** The plot shows the GC content in a 40 bp window centered at the indicated nucleotide position.

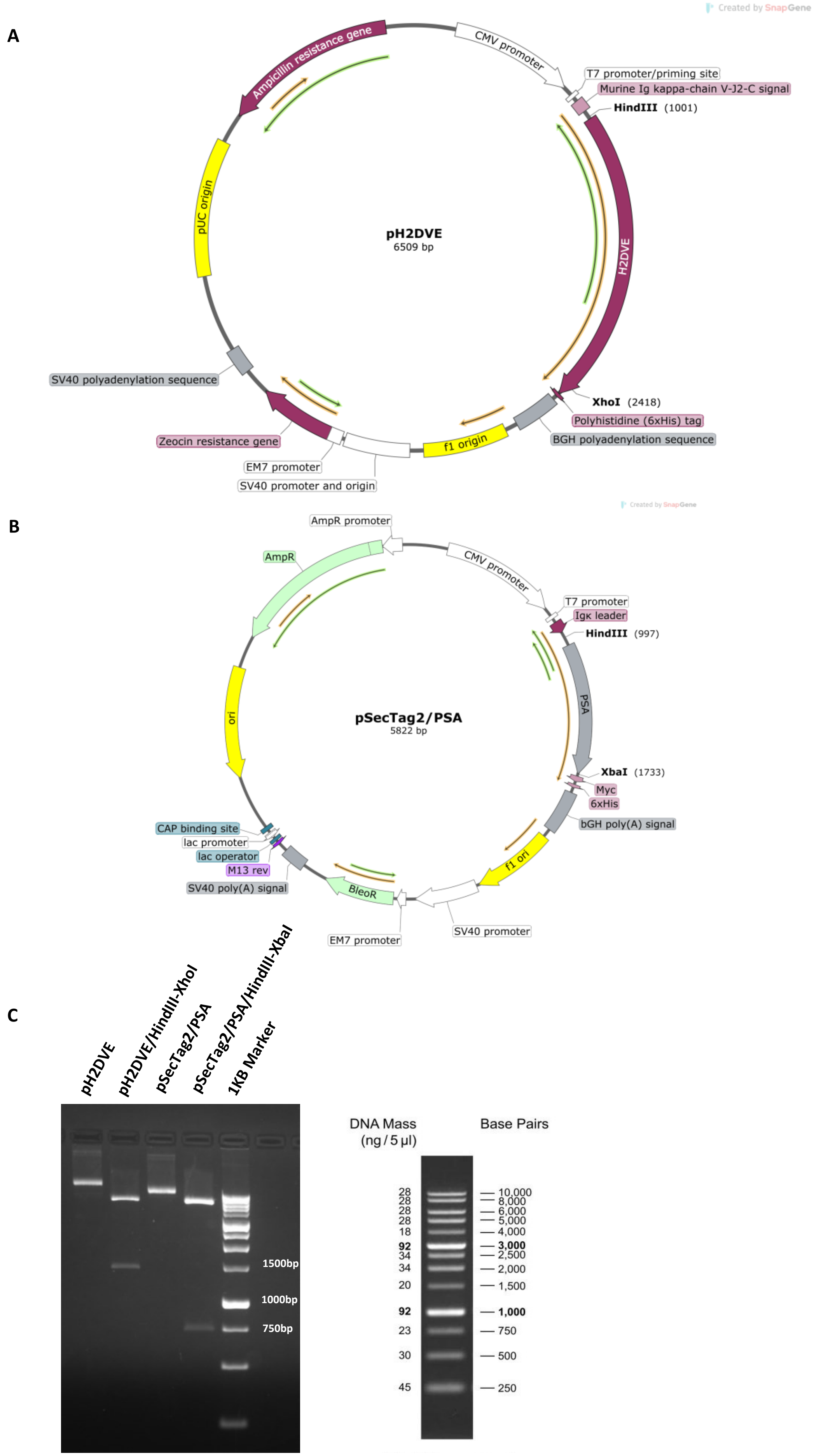

Figure S4: Plasmid sequence map of **(A)** pH2DVE and **(B)** pSecTag2/PSA, created using SnapGene Viewer software (v7.0). **(C)** Electrophoresed DNA of undigested and double restriction digested products of pH2DVE and pSecTag2/PSA, as indicated, where sequence length was estimated using 1 kb molecular marker (GeneDirex).

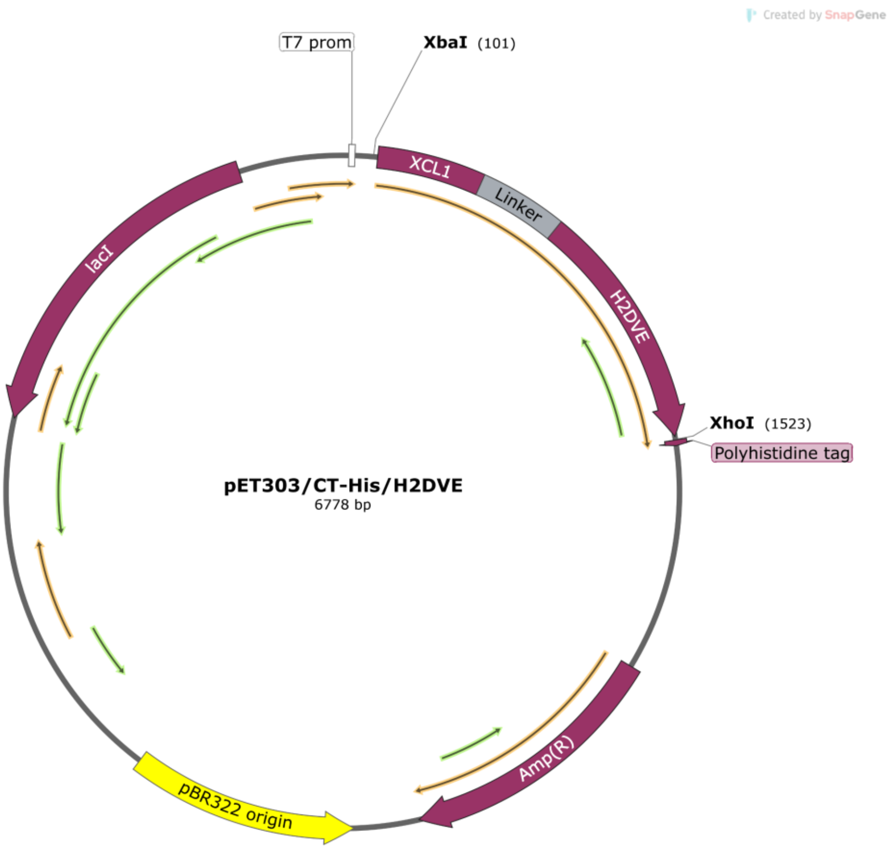

Figure S5: Plasmid sequence map (pET303/CT-His/H2DVE) of the codon-optimized sequence of H2DVE subcloned into the pET303/CT-His vector, created using SnapGene Viewer software (v7.0).
